# Efficacy of pentavalent antimoniate intralesional infiltration therapy for cutaneous leishmaniasis: A systematic review

**DOI:** 10.1371/journal.pone.0184777

**Published:** 2017-09-19

**Authors:** Nayara Castelano Brito, Ana Rabello, Gláucia Fernandes Cota

**Affiliations:** Pesquisas Clínicas e Políticas Públicas em Doenças Infecto-Parasitárias–Centro de Pesquisas René Rachou—Fundação Oswaldo Cruz, Fiocruz, Belo Horizonte, Minas Gerais, Brazil; McGill University Faculty of Education, CANADA

## Abstract

**Background:**

The mainstays of cutaneous leishmaniasis (CL) treatment, in several world regions, are pentavalent antimony (Sb^v^) compounds administered parenterally, despite their recognized toxicity, which requires frequent laboratory monitoring and complicates their use in areas with scarce infrastructure. As result of these drawbacks, the WHO Expert Committee on leishmaniasis has expanded the recommendations for the use of local therapies, including Sb^v^ intralesional infiltration (IL-Sb^v^), as CL therapy alternatives even in the New World. However, the efficacy of these approaches has never been compiled. The aim of this study was to critically and systematically assess the efficacy of IL-Sb^v^ for CL treatment.

**Methodology:**

The PRISMA guidelines for systematic reviews and the Cochrane manual were followed. The sources used were the MEDLINE and LILACS databases and the International Clinical Trials Registry Platform of the World Health Organization. The outcome of interest was a clinical cure, defined as complete re-epithelialization of all lesions. The IL-Sb^v^ pooled cure rate was estimated for several subgroups and direct comparisons were performed when possible.

**Results:**

Thirty nine articles (40 studies) involving 5679 patients treated with IL-Sb^v^ infiltration were included. In direct comparison, only three studies involving 229 patients compared IL-Sb^v^ infiltration *versus* placebo and no difference was observed (OR: 1,9; 95%IC 0,93 to 3,82) based on cure rate 69.6% (95%CI 17.6–96.1%) and 83,2% (95%CI 66–92.7%) for placebo and IL-Sb^v^, respectively. In an alternative and non-comparative analysis, gathering all study arms using the intervention, the pooled IL-Sb^v^ efficacy rate was 75% (95%CI 68–81%). In the Old World, the observed overall IL-Sb^v^ efficacy rate was 75% (95%CI 66–82%), and the cure rates were significantly higher with sodium stibogluconate (SSG) than with meglumine antimoniate (MA): 83% (95%CI 75–90%) *versus* 68% (95%CI 54–79%), p = 0.03. Studies directly comparing IL-Sb^v^ with topical 15% paromomycin ointment, IL hypertonic saline, radiofrequency-induced heat therapy, topical trichloroacetic acid and cryotherapy showed no significant difference in efficacy between the interventions. The analyses suggested a higher efficacy of IL-Sb^v^ combined with cryotherapy (81.8%, 95%IC 62.4–92.4%) when compared with IL-Sb^v^ alone (53.3%, 95%IC 46.1–66%), OR: 3.14 (95%CI 1.1–8.9), p = 0.03. In the New World, the global IL-Sb^v^ efficacy was 77%(95%CI 66–85%). In contrast with the Old World, a significant difference favoring MA in relation to SSG was observed: 61% (95%CI 49–73%) *versus* 82% (95%CI 70–89%).By comparing IL infiltration schedules, it was determined that patients submitted to IL-Sb^v^ treatments longer than 14 days had higher cure rates.

**Conclusions:**

Despite the high heterogeneity and low methodological quality of studies, an indirect comparison shows that the antimony infiltration efficacy rate is similar to that reported for antimony systemic use. The evidence gathered thus far is insufficient to identify the ideal IL therapeutic regime or estimate the rates of adverse events and mucosal late complications.

## Introduction

Cutaneous leishmaniasis (CL) is a non-fatal disease leaving life-long scars and serious disability. Over the past decade, the world wide prevalence and geographical distribution of CL has expanded [1]. The disease is now recognized as a complex and highly variable disease in terms of its epidemiology, etiology, pathology, and clinical features. Furthermore, the large number of reported treatments for CL indicates that no ideal therapy has yet been identified.

The mainstays of treatment for CL in several regions of the world are pentavalent antimony (Sb^v^) compounds administered parenterally. These compounds are used despite their recognized toxicity, which requires frequent laboratory monitoring and complicates the use in areas with scarce resources and infrastructure. The development of a more effective and safer therapeutic alternative for CL is considered a priority. In 2010, the World Health Organization Expert Committee on Leishmaniasis recommended the inclusion of local and topical treatments among the acceptable therapeutic alternatives for New World leishmaniasis [2]. In 2013, the Pan American Health Organization Expert Committee on leishmaniasis also included intralesional treatment in the regional guidelines restricted to reference centers and to single lesions not involving the face or joints [3]. The potential advantages of intralesional infiltration are the use of lower total doses of antimony (and thus less toxic effects) and a more flexible schedule without the requirement of daily drug administration. In addition, antimony therapeutic modality that does not require investment in equipment, which makes it feasible to implement in the short term. However, to the best of our knowledge, the efficacy rate of this approach has never been compiled. The risk of late mucosal complications related to non-systemic treatments, which comprise not only intralesional infiltration but also thermotherapy, cryotherapy and other topical therapies, are also unknown [4].

The aim of this study was to provide a comprehensive and systematic review of the literature, followed by a critical analysis of the available evidence for Sb^v^ intralesional infiltration efficacy in CL.

## Objectives

Our main objective was to assess the efficacy of intralesional Sb^v^ treatment for cutaneous leishmaniasis in New and Old World infections. The secondary objectives were to evaluate whether response to Sb^v^ intralesional treatment is species-dependent or associated with the disease’s geographical distribution, therapeutic schedule or the pentavalent compounds currently available (sodium stibogluconate and meglumine antimoniate).

### Materials and methods

Our review methodology followed the recommendations of the Cochrane Collaboration Group and the recommendations of the PRISMA guidelines [5, 6]. The systematic review was registered in International Prospective Register of Ongoing Systematic Reviews—with initial title: “Efficacy of intralesional pentavalent antimoniate therapy in cutaneous leishmaniasis: a systematic review” getting the record CRD42016038252.Structured searches were conducted in PubMed (MEDLINE), the Cochrane Library, and LILACS using a comprehensive list of key terms that were adapted to each database from inception through September 2016. The PICO question was: **P**opulation: subjects with CL diagnosis; **I**ntervention: antimony intralesional infiltration; **C**omparator, if applicable: any other therapy, placebo or no treatment; **O**utcome: cure rate. Secondary objectives were to assess the adverse events reported with antimony intralesional treatment, to verify whether responses to antimony intralesional therapy are schedule-dependent, species-dependent or associated with the disease geographical distribution (New and Old World), and to determine the rates of relapse and late mucosal involvement after treatment. The International Clinical Trials Registry Platform of the World Health Organization (WHO) was also searched to identify past and ongoing trials using the key word “leishma*”. The reference sections of primary studies as well as narrative and systematic reviews addressing leishmaniasis therapy were examined to search for additional primary studies that might have been missed during the electronic search. The detailed search strategies are described in S1 Text.

The studies were independently selected by two authors (GCF and NCB) and included without publication date, study design or language restrictions. Studies were included if reporting cure rate after intralesional infiltration of pentavalent antimony for CL treatment. Studies involving non-human participants; studies with less than ten participants in the intralesional therapy arm; studies assessing outcome as number of cured lesions (but not cured patients) or addressing intralesional infiltration combined with another therapeutic modality and studies presenting more than 15% of patients lost during follow-up were excluded. All studies matching the inclusion criteria were reviewed by the authors and disagreement on inclusion was settled through consensus checking the information presented in the original studies.

The selected articles were read in full to confirm eligibility and to extract data. Data extraction and quality assessment were carried out by one author and checked by a second reviewer. The reviewers independently extracted data on participant characteristics, predominant parasite species, and interventions and outcomes using a standardized data collection form. The follow-up length and relapse rate were also recorded when available.

The main outcomes studied were clinical cure, defined as complete re-epithelialization of all lesions, and relapse, defined as the reappearance of an active lesion after complete cure. The timing of cure assessment after treatment varied significantly among the studies. Because the time between the end of treatment and healing assessment can potentially influence cure rate, we established three cure rate occasions by pooling studies according to the time at which cure assessment was originally performed and by implementing the current recommendations for outcome measures in CL trials [7]. After pooling the studies, three cure assessment time points counted from the first day of treatment were included in our analysis: 1) epithelialization rate between 30 at 73 days (initial response), 2) 74 at 100 days (initial cure) and 3) 101 at 194 days (definitive cure). To gather as much information as possible, even if the author had defined cure according to clinical assessment performed at a specific moment, information was included in our analysis if available at other moments of interest. The analysis was performed according to the intention-to-treat principle.

We evaluated the quality of the randomized studies using the following criteria: 1) double-blind; 2) concealment of treatment allocation; 3) blinding of outcome assessment; and 4) intention-to-treat analysis. Concealment of treatment allocation was adequate if patients and enrolling investigators could not predict the assignment. Outcome assessment was blinded if the investigator who assessed the outcome had no knowledge of treatment assignment. The Newcastle-Ottawa Scale (NOS)[8] was used to assess the quality of nonrandomized studies. On this scale, each study was measured in three dimensions: 1) selection of study groups, 2) comparability of groups, and 3) determination of the results of interest. For randomized and non-randomized studies, it was assumed inadequate if there was not enough information to assess the quality.

### Quantitative data synthesis

Comprehensive Meta-Analysis® software v.2.2.048 was used to perform one-group meta-analysis of all Sb^v^ intralesional arms by estimating a pooled cure rate for several subgroups of studies. When possible, these estimates were used to indirectly compare groups of interest according to “The Comparing Multiple Interventions Methods Group of the Cochrane Collaboration.” Direct comparisons were performed when available. For all analysis, the intention-to-treat principle was applied, irrespective of how the original study investigators analyzed the data. For dichotomous primary outcomes, the results expressed as odds ratio (OR) and 95% confidence intervals (CI) were calculated using the Mantel–Haenszel random effects model. Random effects model considers presence of between study variation for treatment effect in measurement and estimation of combine effect size. Thus, in the framework of random effects each study has its own population, and random effects of between study parameter variations is added to the previous source of variation, and the various results between studies are examined. So, it can be said that the results from this model are more generalizable than fixed effects model. For the pooled analysis, we calculated the I square (I^2^) statistic, which describes the percentage of total variation across studies attributed to heterogeneity [9]; low, moderate, and high levels of heterogeneity were roughly estimated as I^2^ values of 25%, 50%, and 75%, respectively. Publication bias was assessed using the Trim and Fill method. This algorithm is a non-parametric approach that makes strong assumptions about funnel plot asymmetry and was introduced by Duval and Tweedie [10]. Firstly, the remote and asymmetric part of funnel is removed after estimate of the number of studies in this part (trimming). Then, from the symmetric remaining part is used for estimating of the true center of the funnel. Finally, the removed studies and their missing counterparts are replaced around the center (filling). In the end, estimated true mean and the variance related to it are achieved, based on the completed funnel plot.

## Results

**Fig 1:**
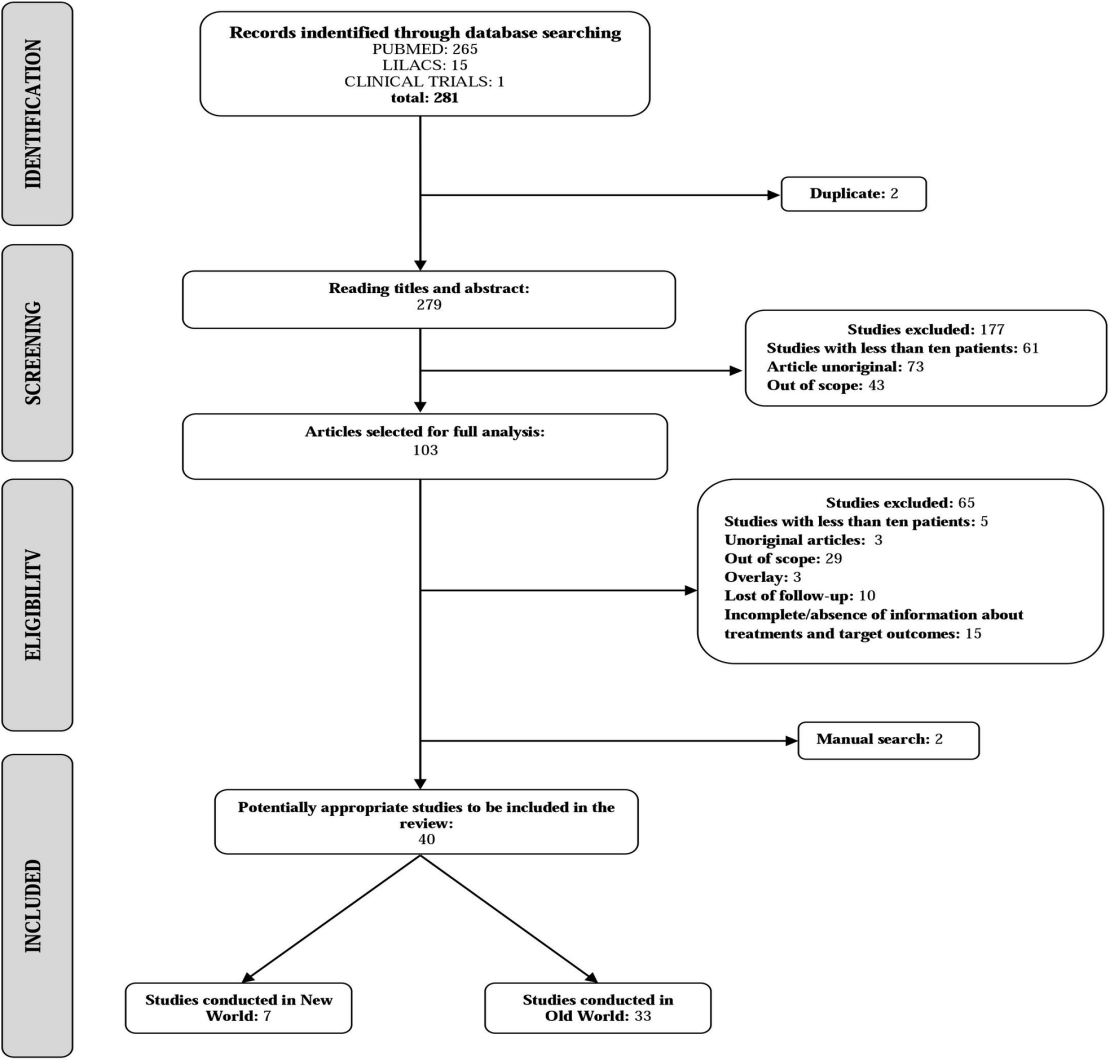
Flow diagram of the study selection process.

The literature search identified 265 articles from PubMed, 15 from LILACS and 1 from ICTRP, in addition to two other titles retrieved from the references of the primary studies. The search flowchart is shown is Fig 1. After exclusion based on titles and abstracts, 103 potentially relevant papers were selected for full text evaluation. Of them, 65 studies were excluded due to the following reasons: the information was incomplete (3); the intralesional arm included fewer than 10 patients (5); Sb^v^ intralesional therapy was not used (out of scope) (29); the same patients were described elsewhere (3); more than 15% of follow-up was lost (10); and the outcome assessment was based on number of cured lesions and not on the number of cured patients (12).

Considering that this criterion of exclusion can be questioned since the number of cured lesions has been defended by some as equivalent to the number of patients cured, details of these 12 studies are presented in S1 Table. Three papers were reviews and were thus excluded. Finally, one study evaluating only patients presenting lupoid leishmaniasis was excluded because it is a disease form with specific therapeutic response [11]. One of the articles described two studies, thus, 39 articles (40 studies)involving 7693 patients, of which 5679 submitted to Sb^v^ intralesional therapy (IL-Sb^v^), were included.

The studies were conducted in endemic CL areas in twelve countries in the Old World (Afghanistan, Iran, Turkey, Sri Lanka, Israel, India, Libya, Morocco, Tunisia, Saudi Arabia, Arab Emirates and Syria) and in three countries in the New World (Bolivian, Brazil and Venezuela). The twenty-seven randomized trials (65%) involved 3322participants and in general used different comparator arms: topical paromomycin (PA), intralesional hypertonic saline (HS), radiofrequency-induced heat therapy (RFHT), topical trichloroacetic acid (TCA), and cryotherapy. We also indirectly compared the efficacy rates by grouping studies by antimony derivative (sodium stibogluconate/SSG *versus* meglumine antimoniate/MA), criterion of cure (clinical *versus* parasitological defined cure) and study design (randomized *versus* non-randomized), in an attempt to address and to explain the heterogeneity among the studies.

Among the non-randomized studies, four were prospective and comparative [12–14], two were prospective with no comparative studies [15, 16] and the remaining eight were retrospective studies with one or more treatment regimen arms reported. In 26 studies (65%), the pentavalent antimony compound used was meglumine antimoniate (MA), while sodium stibogluconate (SSG) was used in other 14studies (33%). One study directly compared the efficacy between SSG and MA [17].

CL was diagnosed if patients had a compatible clinical illness and *Leishmania* was identified in Giemsa-stained smears, culture or biopsy. In a few studies, detection of parasite DNA through the polymerase chain reaction (PCR) was also considered to confirm *Leishmania* infection [13, 18–24].

The definition of cure varied between the studies. Four studies did not report the criteria used [16, 25–27], including one unpublished study whose data were taken from the clinical trials website [28] and another study, published in Farsi, whose information was extracted from the abstract [29]. Most studies defined cure as complete “involution”, for non-ulcerated lesions and “re-epithelialization” of ulcered lesions. In one study, cure was defined by “the onset of a scar at the site of lesion.” [30] Nine of 40 studies (23%) required a negative direct smear to define cure of a clinically healed lesion [19, 31–38], and one study considered only parasitological criteria to define cure [12]. Because of these differences, cure rates will be presented globally and, also, according to the cure criteria adopted in the original studies. Cure rate was originally assessed in the studies at different times after the beginning of treatment (ranging from 1 to 6 months). In most studies, the follow-up was between 6 and 12 months. The methodological characteristics of the studies, namely inclusion, exclusion and cure criteria, are shown in Tables 1 and 2.

**Table 1:** Main methodological characteristics of the Old World leishmaniasis studies.

Year, Author	Country (cases)	Study arms(patients)	Prospective/Comparative	Randomized	Inclusion criteria	Exclusion criteria	Cure criteria for ulcered lesions	Follow-up (months)
**2016, Jaffary**	Iran (90)	MA-IL (30)	Yes/Yes	Yes	Lesion diameter < 3cm, disease duration < 12 weeks, lesion-to-eyelid distance > 2cm, and no history of systemic or topical antileishmanial therapy	Pregnant or lactating, immunosuppressive therapy, and serious side effects of medication.	Complete re-epithelialization plus negative direct skin smear	6
		MA-IL + TCA (30)						
		MA-IL + CO2 (30)						
**2016, Rajabi**	Iran (66)	Azithro (26)	Yes/Yes	Yes	Parasitologically confirmed diagnosis, disease duration of less than 12 weeks, no previous antileishmanial therapy (2 months)	Pregnant or lactating, intolerance or allergy to macrolids, severe cardiovascular, renal, or hepatic diseases	Completere-epithelialization, disappearance of edema, indurations, and other signs of inflammation, plus negative direct skin smear	6
		MA-IL (40)						
**2016, Yesilova**	Turkey (3456)	MA-IL (1728)	No/Yes	No	NR	NR	Complete re-epithelialization	NR
		SSG-IL (1728)						
**2015, Ranawaka**	Sri Lanka(44)	SSG-IL (170)	Yes/Yes	Yes	NR	Pregnant or lactating, history of cardiac, renal, or hepatic disease	Complete re-epithelialization or marked improvement	18
		10% HS (192)						
		15% HS (82)						
**2014, Refai**	Sri Lanka (213)	RFHT (98)	Yes/Yes	Yes	Parasitologically confirmed diagnosis, single lesion	NR	NR	NR
		SSG-IL (115)						
**2014, Stahl**	Afghanistan (87)	SSG-IL (24)	Yes/Yes	Yes	Parasitologically confirmed diagnosis, no previous antileishmanial therapy	Age <12 years, more than one skin lesion, disease duration >3 months, lesions located on eye lids, lips or nose, drug addiction, coinfection with *M*. *tuberculosis* or HIV, and diabetes	Complete re-epithelialization	6
		MWT _DAC N-055_ (31)						
		HF-EC/ MWT _DAC N-055_ (32)						
**2014, Agrawal**	Iran (148)	SSG-IL (58)	No/No	No	NR	NR	Complete re-epithelialization and absence of signs of inflammation plus negative direct skin smear	NR
		RFHT (14)						
		Rifampicin (50)						
		Dapsone (3)						
		Rifampicin + SSG-IL (12)						
		Dapsone+ rifampicin (11)						
**2014, Solomon**	Israel (45)	SSG-IL (21)	No/No	No	NR	NR	Complete re-epithelialization (or for no ulcerative lesions, regression of the lesion)	3
		L-AmB-IV (24)						
**2014, Niforoushzadeh**	Iran (200)	MA-IL (100)	Yes/Yes	Yes	Parasitologically confirmed diagnosis, 6 to 60 years, no previous antileishmanial therapy, lesion diameter < 3cm, disease duration < 12 weeks, lesion-to-eyelid distance > 2cm	Pregnant or lactating	Complete re-epithelialization of the lesion and lack of induration Plus negative direct smear	NR
		MA-IL + TCA (100)						
**2013, Mohammadzadeh**	Iran (136)	MA-IL (38)	Yes/Yes	No	Parasitologically confirmed diagnosis	Pregnant or lactating, patient refusal, underlying conditions or chronic disease	Complete re-epithelialization plus negative direct smear	1
		MA-IM (94)						
		MA-IL + MA-IM (4)						
**2013, Bumb**	India (100)	RFHT (50)	Yes/Yes	Yes	Parasitologically confirmed diagnosis, either gender, aged ≥ 4 years, four or fewer lesions	Lesion size > 5 cm diameter, prior treatment failure with SSG, previous antileishmanial therapy (2 months) underlying chronic diseases	Complete re-epithelialization	18
		SSG-IL (50)						
**2012, Maleki**	Iran (34)	ZnSO4 (24)	Yes/Yes	Yes	Parasitologically confirmed diagnosis; three or fewer lesions; disease duration < 12 weeks, 7 to 60 years, dry cutaneous leishmaniasis	Pregnant or lactating, age < 7 years; lesions on ear, nose, joints and near the eye, previous anti‐leishmaniasis therapy, recurrent infection	Complete re-epithelialization plus negative direct skin smear	2
		MA-IL (10)						
**2012, Niforoushzadeh**	Iran (60)	MA-IL (30)	Yes/Yes	Yes	NR	Pregnant, age < 5 years, palpebral lesions, > 5 lesions, disease duration > 12 week, previous anti‐leishmaniasis therapy, significant underlying diseases	Complete re‐epithelialization flattening of the lesions and lack of indurations plus negative direct smear	6
		RFHT + TCA (30)						
**2012, Safi**	Afghanistan (382)	RFHT (189)	Yes/Yes	Yes	Parasitologically confirmed diagnosis, age of > 5 years, single lesion	Previous anti‐leishmaniasis therapy, lesion-to-eyelid distance < 2 cm or on the lips or nose	Complete re-epithelialization with no inflammation and resolution of the papule and/or nodule.	6
		MA-IL (193)						
**2011, Layegh**	Iran (110)	L- AmB-IL (50)	Yes/Yes	Yes	Parasitologically confirmed diagnosis, disease duration < 6 month, no previous antileishmanial therapy (3 months)	Pregnant or lactating, taking any other specific treatment while participating in the study, previous anti‐leishmaniasis therapy (2 months), significant underlying disease such as cardiac, renal, or liver dysfunction	Complete re-epithelialization	6
		MA-IL (60)						
**2010, van Thiel**	Afghanistan (163)	SSG-IL (118)	No/No	No	NR	NR	Complete re-epithelialization	6
		SSG-IL + Cryo (45)						
**2010, Bumb**	India (220)	SSG-IL 1x/w (110)	Yes/Yes	Yes	Parasitologically confirmed diagnosis, typical papules, nodules or plaques	Age < 5 years, pregnant women, >2 lesions, underlying systemic disease, previous anti‐leishmaniasis therapy	Complete re-epithelialization	24
		SSG-IL 2x/w (110)						
**2010, Ranawaka**	Sri Lanka (154)	SSG-IL (87)	Yes/Yes	Yes	NR	NR	Complete re-epithelialization without any palpable lesions	18
		7% HS (67)						
**2009, Solomon**	Israel (54)	SSG-IL (33)	No/No	No	NR	NR	Complete re-epithelialization (or flattened of skin lesions), or reduction of the lesion to less than 3 mm, and no relapse in 3 months	3
		SSG-IV (21)						
**2009, Layegh**	Iran (79)	MA-IL (39)	Yes/Yes	Yes	Parasitologically confirmed diagnosis, age ≤ 13 years, disease duration < 12 weeks	Age > 13 years, disease duration > 3 months, allergy to antimonial drugs, any other therapeutic method use	Complete re-epithelialization; disappearance other signs of inflammation plus a negative direct skin smear	6
		Cryo (40)						
**2008, Qasmi**	Morocco (12)	MA-IL (12)	No/No	No	Age < 16 years	NR	NR	6
**2007, Nilforoushzadeh**	Iran (90)	MA-IL + honey (45)	Yes/Yes	Yes	Parasitologically confirmed diagnosis, no previous antileishmanial therapy, absence of malnutrition, cardiac, renal or hepatic disease or another contraindication	Pregnant and lactating	Complete re-epithelialization and disappearance of the induration	4
		MA-IL (45)						
**2007, Sadeghian**	Iran (117)	RFHT (57)	Yes/Yes	Yes	NR	Pregnant, age < 5 years, facial lesions, previous antileishmanial therapy, significant underlying diseases	Complete re-epithelialization (lesions flattened, no induration, and epidermal creases appeared)	6
		MA-IL (60)						
**2006, Niforoushzadeh**	Iran (80)	MA-IL (40)	Yes/Yes	Yes	NR	Pregnant and lactating, previous antileishmanial therapy, underlying diseases, history of allergy to MA, palpebral lesions, >5 lesions or lesion > 3 cm diameter, > disease duration > 12 weeks	Complete re-epithelialization	3
		TCA (40)						
**2006, Salmanpour**	Iran (60)	MA-IL (20)	Yes/Yes	Yes	NR	NR	NR	NR
		Cryo (20)						
		Cryo + MA-IL (20)						
**2006, Sadeghian**	Iran (72)	HS (36)	Yes/Yes	Yes	Parasitologically confirmed diagnosis, either gender, aged ≥ 5 years, contraindication to systemic therapy	Pregnant, facial lesions or lesions on joint, sporotrichoid, lupoid or erysipeloide leishmaniasis type, underlying chronic diseases	Complete re-epithelialization with no signs inflammation plus a negative direct skin smear	6
		MA-IL (36)						
**2005, Shazad**	Iran (60)	Paro (30)	Yes/Yes	Yes	1–3 lesions, lesion diameter < 5, disease duration < 3 months, no previous antileishmanial therapy, no allergy to paromomycin	NR	Complete re-epithelialization	6
		MA-IL (30)						
**2003, Niforoushzadeh**	Iran (73)	TCA (38)	Yes/Yes	Yes	NR	NR	NR	NR
		MA-IL (35)						
**2003, Faghihi**	Iran (96)	Paro (48)	Yes/Yes	Yes	NR	Pregnant, >3 lesions, ulcerative lesions, lesions with cartilage or lymphatic involvement or hypersensitivity to the drugs	Complete re-epithelialization and return to normal tissue texture in less than 2 months, with no residual scar or relapse	12
		MA-IL (48)						
**2001, Salmanpour**	Iran (96)	Keto (64)	Yes/Yes	Yes	NR	Pregnant or lactating, age < 3 years, concomitant renal, liver or heart disease	Complete re-epithelializationwith little or no scarring	6
		MA-IL (32)						
**1999, Chahed**	Tunisia (109)	MA-IL (52)	Yes/Yes	Yes	No previous antileishmanial therapy	Pregnant and lactating, underlyingchronic disease, contraindications to Glucantime use	Complete re-epithelialization	2
		Éosine (57)						
**1990, el Darouti**	Arab Emirates (44)	Cryo + MA-IL (15)	Yes/Yes	No	NR	NR	NR	3
		Cryo (14)						
		SSG-IL (15)						
**1979, Ghosn**	Syria (31)	SSG-IL (19)	No/No	No	NR	NR	Complete re-epithelialization and a negative direct skin smear	3
		SSG-IM (12)						

**NR:**no reported **Azithro:**Azitromycin **Cryo:** Cryotherapy **CO2:**Carbon Dioxide Laser **HS:** intralesional hypertonic saline **Keto:** Ketoconazole **Paro:** Paromomycin **L-AmB-IL:** intralesional liposomal amphotericin B **MA-IL:** intralesional meglumine antimoniate **MA-IM:** intramuscular meglumine antimoniate **MWT DAC N-055**: moist wound treatment (MWT) with 0.045% pharmaceutical chlorite(DAC N-055) **RFHT:** radiofrequency heat therapy **RFHT/MWT DAC N-055:** high-frequency(HF)-electro-cauterization (EC) combined with subsequent moist treatment(MWT) with 0.045% pharmaceutical sodiumchlorite solution **SSG-IL:** intralesional sodium stibogluconate **TCA:** topical trichloroacetic acid **ZnSO4:**Intralesional zinc sulfate

**Table 2:** Main methodological characteristics of the New World leishmaniasis studies.

Year, Author	Country (cases)	Study arms (number of patients)	Prospective/Comparative	Randomized	Inclusion criteria	Exclusion criteria	Cure criteria for ulcered lesions	Follow-up (months)
**2016,da Silva**	Brazil (31)	MA-IL (31)	No/No	No	Parasitologically confirmed diagnosis, patients presenting relapse or not treated disease or contraindication or toxicity with systemic use of MA	NR	Complete re-epithelialization of the ulcer, without any induration of the lesion site	NR
**2016,Soto A**	Bolivian (90)	SSG 3-IL (30)	Yes/Yes	Yes	Either gender, > 12 years, parasitologically confirmed diagnosis, no previous antileishmanial therapy, no mucosal lesions, no history of concomitant diseases	NR	No doubling in size at 1 month, at least 50% diminution in size at 3 months, and complete re-epithelialization at 6 months	6
		SSG 5-IL (30)						
		Penta120-IL (30)						
**2016,Soto B**	Bolivian (60)	SSG 5-IL (30)	Yes/Yes	Yes	Either gender, > 12 years, parasitologically confirmed diagnosis, no previous antileishmanial therapy, no mucosal lesions, and no history of concomitant diseases	NR	No doubling in size at 1 month, at least 50% diminution in size at 3 months, and complete re-epithelialization at 6 months.	6
		Penta 240-3-IL (30)						
**2013,Soto**	Bolivian (80)	MA-IL (30)	Yes/Yes	Yes	Parasitologically confirmed diagnosis, > 12 years, one ulcerative lesion, lesion area < 900 mm^2^	Previous antileishmanial therapy (< 3 months), mucosal lesions, concomitant diseases	No doubling in size at 1 month, at least 50% diminution in size at 3 months, and complete re-epithelialization at 6 months.	6
		Cryo (20)						
		placebo cream (30)						
**2012,Vasconcellos**	Brazil (24)	MA-IL (24)	No/No	No	Parasitologically confirmed diagnosis, no previous antileishmanial therapy, and presence of contraindication to systemic use of MA	NR	Complete re-epithelialization	12
**1997, Oliveira-Neto**	Brazil (74)	MA-IL (74)	Yes/No	No	Parasitologically confirmed diagnosis, single or few ulcerative lesions and presence of contraindication to systemic use of MA	NR	Complete re-epithelialization and no reactivation of lesions or development of mucosal lesions during follow-up.	>24
**1995,Yépez,**	Venezuela (89)	MA-IL (30)	Yes/Yes	No	NR	Facial or digital lesions, secondary infections with satellite adenopathy	Lesion showed a scar	12
		MA-IL+ lidocaine (29)						
		lidocaine (30)						
**1995, Gadelha**	Brazil (64)	MA-IL (64)	Yes/No	No	Single or few and small lesions in patients presenting relapse or lesions not cured with MA and/or pentamidine	NR	NR	NR

**NR:** no reported **Cryo:** Cryotherapy **MA-IL:** intralesional meglumine antimoniate **Penta120-IL:** intralesional pentamidine (120 mg/mm^2^ of lesion area) for 3 injections **Penta 240-3-IL:** intralesional pentamidine (240 mg/mm^2^ of lesion area) in 3 injections **SSG-IL:** intralesional sodium stibogluconate **SSG 3-IL:** intralesional sodium stibogluconate for 3 injections **SSG 5-IL:** intralesional sodium stibogluconate in 5 injections

The patient characteristics varied according with geographical location. In the Old World leishmaniasis studies, both children and adults were included; in the Americas, no studies evaluated only children. The proportion of men and women was balanced in both Old and New World studies. In relation to the disease presentation, most studies predominantly recruited patients with few and small ulcerated lesions. In the Old World studies, the most affected body site was the head, in contrast to the Americas, where most lesions were on the limbs (Tables 3 and 4).

**Table 3:** Characteristics of the population enrolled in the Old World leishmaniasis studies.

Year, Author	Age (mean, years ± SD)	Gender male/female	Mean of lesions per patient ± SD	Lesion site^a^ ^b^	Mean of lesion area/mm2 ± SD	Leishmania species characterization (n)	Type of lesion (number of lesions)	Mean of lesion duration (months before therapy ± SD)
**2016, Jaffary**	24.5 ± 14	NR	NR	Upper limbs (43), lower limbs (15), face (8), trunk (5), neck (4), more than one site (15) a	NR	NR	Nodule (66), papule (48)	NR
**2016, Rajabi**	Azitro: 21.2 ± 16.4	Original table with error	1.5	Head/neck (29), upper limbs (54), lower limbs (14), trunk (2)b	147±239	NR	Papule (32), plaque (28), ulcer (10), nodule (24)	NR
	MA-IL: 30.0 ± 17.3				254±364			
**2016, Yesilova**	MA-IL: 22.6 ± 17.8	1487/1969	NR	Head/neck (1665), upper limbs (347), lower limbs (1225), trunk (18), mucosal (94), generalized (98) a	NR	NR	Ulcer (1891), papule (240), nodule (1233)	More than 6 weeks in 88% of the patients
	SSG-IL: 25,4 ± 16,5							
**2015, Ranawaka**	32.7	286/158	1.6	Face (98), upper limbs (252), lower limbs (116), trunk (54)b	NR	NR	Papule (181), nodule (56), plaque (31), ulcer (271)	NR
**2015, Refai**	NR	NR	NR	NR	NR	NR	NR	NR
**2014, Stahl**	29	32/37	NR	Head (10), trunk (1), upper limbs (42), lower limbs (16) a	NR	*L*. *major (16)*	NR	NR
						*L*. *tropica* (28)		
**2014, Agrawal**	3.3 ± 1.4	92/59	1.4	Face (109), lower limbs (12), upper limbs (15), trunk (2), multiple sites (13) a	NR	*L*. *tropica* (13)	Plaque (164), papule (53)	4.5 ± 0.29
**2014, Solomon**	8.8	31/16	2.8	Head/neck (36), trunk (9), upper limbs (16), lower limbs (1)^**a**^	NR	NR	NR	3.8
**2014, Nilforoushzadeh**	10.7 ± 22	101/86	1.6	NR	359 ± 55.6	NR	Papule (20), nodule (71), plaque (139), ulcer (89)	NR
**2013, Mohammadzadeh**	30.8 ± 20.1	76/61	2.2 ± 2.1	NR	NR	NR	NR	NR
**2013, Bumb**	RFHT: 20	47/53	1.6	NR	NR	*L*. *tropica* (27)	NR	3
	SSG-IL: 20.5							3
**2012, Maleki**	NR	NR	NR	NR	NR	NR	NR	NR
**2012, Nilforoushzadeh**	25.1 ± 13.3	NR	1.3	Head and upper limbs (61), lower limbs (15)^**a**^	MA-IL:32 ± 9	NR	NR	NR
					RFHT+ TCA: 27 ± 4			
**2012, Safi**	RFHT: 14	177/205	NR	Face/Neck (161), upper limbs (26), lower limbs (2)^**a**^	100	NR	Ulcer (6), nodule (168), papule (208)	NR
	MA-IL: 13				200			
**2011, Layegh**	MA-IL: 25.3 ± 15.7	44/66	1.4 ± 0.8	Head/necks (48), upper limbs (50), leg/trunk (12)^**a**^	NR	NR		1 ± 0.4
	LAmB-IL:20.5 ± 18,7		1.9 ± 1					1.1 ± 0.3
**2010, van Thiel**	NR	161	1.3	Hands/neck/head (20), upper limbs (61), trunk/lower limbs (132)^b^	NR	NR	Ulcer (64), nodule (57)	0.9
**2010, Bumb**	NR	104/116	1.4	Face (88), limbs (197), trunk (13)b	NR	L. tropica (18)	Papule (110), plaque (188)	Half of the patients had lesions with < 3 months duration, most of the rest were between 3 and 6 months
**2010, Ranawaka**	32	99/55	1.5	Ear (3), nose (3), lip (5), face (30), upper limbs (123), lower limbs (43), trunk (14), buttock (1)^**a**^	NR	NR	Papule (52), nodule (28), plaque (14), ulcer (121)	7.2
**2009, Solomon**	23 ± 9	44/10	6	NR	NR	NR	NR	SSG-IL: 2.5
								SSG-IV: 4
**2009, Layegh**	Cryo: 6.8 ± 3.4	38/41	1.9 ± 1.2	Head/necks (55), hand (23), foot (7)b	NR	NR	Papule (72), nodule (5), ulcer (3)	2.8 ± 2.9
	MA-IL: 6.2 ± 3.4		1.4 ± 0.8					3 ± 4.5
**2008, Qasmi**	12	02/out	1	Face (9), neck (3), upper limbs (1)^**a**^	NR	NR	Ulcer: (8), nodule: (4)	11.8
**2007, Nilforoushzadeh**	Honey+MA-IL:26.1 ± 15.1	61/29	1.3 ± 0.7	Foot (42), hand (37), other areas (11) b	NR	NR	Plaque (62),nodule (17), papule (7), ulcer (4)	NR
	MA-IL: 25.6 ± 14.9		1.7 ± 0.7					
**2007, Sadeghian**	RFHT: 25.1 ± 13	66/51	1.5	Upper limbs (77), lower limbs (57), trunk (43)^b^	NR	NR	Papule (55), plaque (56), nodule (66)	1.1 ± 0.5
	MA-IL: 22.6 ± 12							0.9 ± 0.5
**2006, Niforoushzadeh**	25.7	40/33	1.3	NR	NR	NR	Papule (44), nodule (23), plaque (12), ulcer (6)	1.3
**2006, Salmanpour**	NR	NR	NR	NR	NR	NR	NR	NR
**2005, Sadeghian**	HS:18.7 ± 2	NR	NR	NR	NR	NR	NR	NR
	MA-IL: 20.5 ± 3							
**2005, Shazad**	Paro: 20.6 ± 1.2	60	2.3	Head/neck (25), upper limbs (61), lower limbs (47), trunk (3)^b^	NR	NR	Papule (23), nodule (14), ulcer (99)	1.3 ± 0.1
	MA-IL: 21.7 ± 2							1.3 ± 0.2
**2003, Nilforoushzadeh**	NR	NR	NR	NR	NR	NR	NR	NR
**2003, Faghihi**	16	40/56	2	NR	NR	NR	NR	NR
**2001, Salmanpour**	Keto: 20.7± 12.7	44/52	2.5	NR	NR	NR	NR	2.6 ± 1.4
	MA-IL: 21.3 ± 14.7		2.3					3.1 ± 1.4
**1999, Chahed**	NR	56/53	2.5 ± 0.2	Upper limbs (19), face (19), lower limbs (45), trunk (2)^**a**^	NR	NR	Papule (14), nodule (9), ulcer (49), plaque (13)	1.76 ± 0.16
**1990, el Darouti**	Cryo+MA-IL: 32	25/19	1.1	Lower limbs (28), upper limbs (22), face (8), trunk (2)^b^	NR	NR	NR	2.2
	Cryo:30.5							
	SSG-IL: 32.5							
**1979, Ghosn**	23.8	22/set	NR	NR	NR	NR	Ulcer (13), nodule (21)	NR

**NR:** no reported **Azithro:** Azitromycin **Cryo:** Cryotherapy **HS:** intralesional hypertonic saline **Keto:** Ketoconazole **Paro:** Paromomycin **L-AmB-IL:** intralesional liposomal amphotericin B **MA-IL:** intralesional meglumine antimoniate **RFHT:** radiofrequency heat therapy **SSG-IL:** intralesional sodium stibogluconate **TCA:** topical trichloroacetic acid

^**a**^: number of patients

^**b**^: number of lesions

**Table 4:** Characteristics of the population enrolled in the New World leishmaniasis studies.

Year, Author	Age (mean, years ± SD)	Gender male/female	Number of lesions per patient (mean ± SD)	Lesion site^a^^,^ ^b^	Mean of lesion area/mm^2^± SD	Leishmania species characterization (n)	Type of lesion	Mean of lesion duration (months before therapy ± SD)
**2016, da Silva**	63	15/16	1.4	Head/neck: (10), upper limbs: (10), leg: (7), trunk: (4) ^a^	170	NR	Ulcer: (17), papule: (8)	NR
**2016, Soto A**	29 ± 11	NR	NR	NR	310 ± 120	*L*. *braziliensis* (17); *L*. *amazonensis* (1); *L*. *lainsoni* (2), *L*. *guyanensis* (1)	NR	NR
**2016, Soto B**	27 ± 7	NR	NR	NR	260 ± 135	*L*. *braziliensis* (13) *L*. *species* (2)	NR	NR
**2013, Soto**	29 ± 12	NR	NR	Upper limbs (17), head/neck (11), trunk (2), lower limbs (49)^b^	218 ± 160	*L*. *braziliensis* (36); *L*. *guyanensis* (2); *L*. *amazonensis* (2); *L*. *lainsoni* (2)	NR	NR
**2012, Vasconcellos**	64	10/14	1 §	Lower limbs (8) and other sites (15)^b^	NR	NR	NR	3
**1997, Oliveira-Neto**	25.8 ± 12.7	44/30	1.1 ± 0.39	NR	51.4 ± 23.9	*L*. *braziliensis* (15)	NR	2.5 ± 1
**1995, Yépez**	25	55/51	1.4	Trunk: (16), upper limbs: (48), lower limbs: (59), feet: (7) ^a^	NR	NR	NR	NR
**1995, Gadelha**	NR	38/26	1.6	Lower limbs (43), upper limbs (14), trunk (8), head (8), not defined (3)^b^	NR	NR	NR	NR

**NR:** no reported

^**a**^: number of patients

^**b**^: number of lesions

*Leishmania* species characterization was achieved in only seven studies (18%) [13, 15, 18, 19, 21, 39, 40]. *L*. *braziliensis* and *L*. *tropica* were the most reported species in the New World and Old World studies, respectively. The summarized cure rates according to the intention-to-treat analysis are shown in Tables 5 and 6.

**Table 5:** Outcomes in Old World leishmaniasis studies.

Year, Author	Study arms (number of patients)	Epithelialization rate between 30–73 days, number of patients (%)	Epithelialization rate between 74–100 days, number of patients (%)	Epithelialization rate between 101–194 days, number of patients (%)	Follow-up lost	Relapse after cure /n
**2016, Jaffary**	MA-IL (30)	10 (38.5)	-	-	14/90	0/76
	MA-IL + TCA (30)	27 (90)				
	MA-IL + CO2 (30)	27 (90)				
**2016, Rajabi**	Azithro (26)	20/26 (77.8)	-	-	2/66	0/64
	MA-IL (40)	30/40 (76.3)				
**2016, Yesilova**	MA-IL (1728)	1668/1728 (96.5)	-	-	-	NR
	SSG-IL (1728)	1574/1728 (90.5)				
**2015, Ranawaka**	SSG-IL (170)	158/170 (92.9)	-	-	6/170	0/427
	10% HS (192)	178/192 (89.1)			8/192	
	15% HS (82)	74/82 (90.2)			3/82	
**2014, Refai**	RFHT (98)	57/98 (58.5)	69/98 (70.5)	-	NR	NR
	SSG-IL (115)	60/115 (52.3)	81/115 (70.5)			
**2014, Stahl**	SSG-IL (24)	15/24 (62.5)	-	-	1/24	NR
	MWT DAC N-055 (29)	23/29 (79.3)			6/29	
	HF-EC/MWT DAC N-055 (28)	20/28 (71.4)			5/28	
**2014, Agrawal**	SSG-IL (58)	49/58 (84.4)	-	-	9/148	NR
	RFHT (14)	13/14 (91.8)				
	Rifampicin (50)	41/50 (82)				
	Dapsone (3)	2/3 (66.7)				
	Rifampicin + SSG-IL (12)	11/12 (90.1)				
	Dapsone+ Rifampicin (11)	10/11 (90.1)				
**2014, Solomon**	SSG-IL (21)	-	14/21 (66.6)	-	0/45	0/45
	L-AmB-IV (24)		18/24 (75)			
**2014, Nilforoushzadeh**	MA-IL (100)	76/100 (76)	-	-	4/100	NR
	MA-IL + TCA (100)	78/100 (78)			9/100	
**2013, Mohammadzadeh**	MA-IL (38)	31/38 (81.6)	-	-	18/155	NR
	MA-IM (94)	72/94 (75.8)				
	MA-IL + MA-IM (4)	4/4 (100)				
**2013, Bumb**	RFHT (50)	25/50 (50)	49/50 (98)	49/50 (98)	0/50	0/50
	SSG-IL (50)	28/50 (56)	43/50 (93)	47/50 (94)	0/50	0/50
**2012, Maleki**	ZnSO4 (24)	8/24 (33.3)	-	-	11/45	NR
	MA-IL (10)	8/10 (80)				
**2012, Nilforoushzadeh**	MA-IL (30)	-	-	24/30 (63.2)	0/60	NR
	RFHT + TCA (30)			16/30 (42.1)		
**2012, Safi**	RFHT (189)	-	-	143/193 (74)	NR	NR
	MA-IL (193)			156/189 (82.5)		
**2011, Layegh**	L- AmB (50)	-	-	22/50 (44)	NR	NR
	MA-IL (60)			29/60 (48.3)		
**2010, van Thiel**	SSG-IL (118)	-	-	65/118 (55.1)	NR	18/118
	SSG-IL + Cryo (45)			30/45 (66.7)		5/45
**2010, Bumb**	SSG-IL 1x/w (110)	68/110 (62)	-	101/110 (92)	22/220	0/220
	SSG-IL 2x/w (110)	80/110 (73)		106/110 (96)		
**2010, Ranawaka**	SSG-IL (87)	-	87/87 (100)	NR	NR	0/154
	7% HS (67)		62/67 (92.2)			
**2009, Solomon**	SSG-IL (33)	-	30/33 (91)	-	0/54	NR
	SSG-IV (21)		18/21 (86)			
**2009, Layegh**	MA-IL (39)	-	-	10/39 (25%)	3/39	NR
	Cryo (40)			31/40 (52.5)	3/40	
**2008, Qasmi**	MA-IL (13)	8/13 (61.5)	-	-	1/13	0/13
**2007, Nilforoushzadeh**	MA-IL + honey (45)	32/45 (71.1)	-	32/45 (71.1)	10t/90	NR
	MA-IL (45)	23/45 (51.1)		24/45 (52)		
**2007, Sadeghian**	RFHT (57)	46/57 (80.7)	-	-	0/117	0/117
	MA-IL (60)	34/60 (56.7)				
**2006, Nilforoushzadeh**	MA-IL (40)	23/40 (57.5)	-	-	5/40	4/35
	TCA (40)	26/40 (65)			2/40	5/38
**2006, Salmanpour**	MA-IL (20)	15/20 (75)	-	-	NR	NR
	Cryo (20)	13/20 (67.8)				
	Cryo + MA-IL (20)	18/20 (89)				
**2005, Sadeghian**	HS (36)	9/36 (25)	-	9/36 (25)	1/36	NR
	MA-IL (36)	15/36 (52)		15/36 (52)		
**2005, Shazad**	Paro (30)	20/29 (69)	-	-	1/30	0/56
	MA-IL (30)	8/27 (67)			3/30	
**2003, Nilforoushzadeh**	TCA (38)	26/38 (68)	-	-	7/80	NR
	MA-IL (35)	23/35 (65.7)				
**2003, Faghihi**	Paro (48)	8/48 (16.6)	-	-	0/96	NR
	MA-IL (48)	20/48 (41.7)				
**2001, Salmanpour**	Keto (64)	57/64 (89)	-	-	0/96	NR
	MA-IL (32)	23/32 (72)				
**1999, Chahed**	MA-IL (52)	22/52 (41.7)	43/52 (83.3)	-	NR	NR
	Eosine (57)	24/57 (41.7)	47/57 (83.3)			
**1990, el Darouti**	Cryo + MA-IL (15)	15/15 (100)	-	-	0/44	NR
	Cryo (14)	11/15 (68)				
	SSG-IL (15)	6/15 (44)				
**1979, Ghosn**	SSG-IL (16)	11/16 (68.7)	-	-	NR	NR
	SSG-IM (12)	12/12 (100)				

**NR:** no reported **Azithro:** Azitromycin **Cryo:** Cryotherapy **CO2:**Carbon Dioxide Laser **HS:** intralesional hypertonic saline **Keto:** Ketoconazole **Paro:** Paromomycin **L-AmB-IL:** intralesional liposomal amphotericin B **MA-IL:** intralesional meglumine antimoniate **MA-IM:** intramuscular meglumine antimoniate **MWT DAC N-055:** moist wound treatment (MWT)with 0.045% pharmaceutical sodiumchlorite solution(DAC N-055) **RFHT:** radiofrequency heat therapy **RFHT/MWT DAC N-055:** high-frequency(HF)-electro-cauterization (EC) combined with subsequent moist wound treatment (MWT) with 0.045% pharmaceutical sodium chlorite solution **SSG-IL:** intralesional sodium stibogluconate **TCA:** topical trichloroacetic acid **ZnSO4:** intralesional zinc sulfate

**Table 6:** Outcomes in New World leishmaniasis studies.

Year, Author	Study arms (number of patients)	Epithelialization rate between 30–73 days, number of patients (%)	Epithelialization rate between 74–100 days, number of patients (%)	Epithelialization rate between 101–194 days, number of patients (%)	Follow-up lost	Relapse after cure
**2016, da Silva**	MA-IL (31)	-	21/31	22/31	-	-
**2016, Soto A**	SSG 3-IL (30)	4/30 (13.3)	17/30 (56.6)	-	1/30	2/30
	SSG 5-IL (30)	11/30 (36.6)	20/30 (66.6)	-	-	2/30
	Penta-120-IL (30)	12/30 (40)	20/30 (66.6)	21/30 (70)	1/30	0/30
**2016, Soto B**	SSG 5-IL (30)	3/30 (10)	16/30 (53.3)	20/30 (66.6)	-	NR
	Penta-240-3-IL (30)	11/30 (36.6)	22/30 (73.3)	-	1/30	-
**2013, Soto**	MA-IL (30)	9/30 (30)	21/30 (70)	-	1/30	-
	Cryo (20)	1/20 (5)	4/20 (20)		1/30	-
	Placebo cream (30)	2/30 (6.6)	5/30 (16.6)		1/30	2/30
**2012, Vasconcellos**	MA-IL (24)	-	20/24 (83.3)	-	0/24	NR
**1997, Oliveira-Neto**	MA-IL (74)	-	59/74 (79.7)	-	3/74	0/56
**1995, Yepéz**	MA-IL (30)	25/30 (83.3)	-	-	1/30	NR
	MA-IL+ Lidocaine (29)	29/29 (100)			0/29	
	Lidocaine (30)	28/30 (93.3)			1/30	
**1995, Gadelha**	MA-IL (64)	60/64 (93.7)	-	-	3/64	NR

**NR:** no reported **Cryo:** Cryotherapy **MA-IL:** intralesional meglumine antimoniate **Penta120-IL:** intralesional pentamidine (120 mg/mm^2^ of lesion area) for 3 injections **Penta 240-3-IL:** intralesional pentamidine (240 mg/mm^2^ of lesion area) in 3 injections **SSG-IL:** intralesional sodium stibogluconate **SSG 3-IL:** intralesional sodium stibogluconate for 3 injections **SSG 5-IL:** intralesional sodium stibogluconate in 5 injections

Therapeutic regimens varied widely among studies. In the Old World leishmaniasis studies, the number of infiltrations ranged from 1–16, and the most common interval was seven days (Table 7). In the New World, the studies presented a maximum of 10 infiltrations, performed at intervals ranging from one to fifteen days (Table 8).

**Table 7:** MA intralesional therapy schedules among Old World leishmaniasis studies.

**Year, (Author)**	**Country (cases)**	**Number of infiltrations**	**Interval between infiltrations (days)**	**Treatment duration (interval between the first and the last infiltration in days)**
**2016, Jaffary**	Iran	Mean 13.6	2 (twice a week)	Maximum 32
**2016, Rajabi**	Iran	8	7	56
**2016, Yesilova**	Turkey	8	2 (twice a week)	28
**2015, Ranawaka**	Sri Lanka	3.7	7 days between the first 3 injections; 14 days between the fourth and fifth injections; then every 30 days until cure	Up to 109
**2015, Refai**	Sri Lanka	Up to 10	7	Up to70
**2014, Stahl**	Afghanistan	NR	daily treatments (with the exception of Fridays) during the first week, twice a week until the end of week 4and thereafter once a week until wound closure	28
**2014, Agrawal**	Iran	5 to 7	2 (twice a week) or 7	NR
**2014, Solomon**	Israel	Mean 3	every 21 to 28	NR
**2014, Niforoushzadeh**	Iran	Up to 8	2 (twice a week)	28
**2013, Mohammadzadeh**	Iran	6 to 15	2 (twice a week)	Up to 56
**2013, Bumb**	India	7	2 (twice a week)	28
**2012, Maleki**	Iran	6	7	42
**2012, Niforoushzadeh**	Iran	Up to 16	2 (twice a week)	56
**2012, Safi**	Afghanistan	5	7	35
**2011, Layegh**	Iran	Up to 8	7	56
**2010, van Thiel**	Afghanistan	Median 5	1 at 3	Up to 56
**2010, Bumb**	India	5 to 7	7	49
**2010, Ranawaka**	Sri Lanka	NR	7 days between the first 3 injections; 14 days between the fourth and fifth injections; then every 30 days until cure	NR
**2009, Solomon**	Israel	mean 2,5	every 21 to 28	Mean 53
**2009, Layegh**	Iran	Mean 4	NR	42
**2008, Qasmi**	Morocco	3 to5	7	Up to 35
**2007, Nilforoushzadeh**	Iran	Up to 6	7	Up to 42
**2007, Sadeghian**	Iran	4	7	28
**2006, Nilforoushzadeh**	Iran	Up to 6	7	42
**2006, Salmanpour**	Iran	6 to 8	7	42 to56
**2006, Sadeghian**	Iran	6 to 10	7	42 to70
**2005, Shazad**	Iran	10	1	20
**2003, Niforoushzadeh**	Iran	6	7	42
**2003, Faghihi**	Iran	Up to 12	7	Up to 90
**2001, Salmanpour**	Iran	6 to 8	15	120
**1999, Chahed**	Tunisia	NR	2 (twice a week)	NR
**1990, el Darouti**	Arab Emirates	10	1	20
**1979, Ghosn**	Syria	Mean 3.4	7	Up to 28

**NR:** no reported

**Table 8:** Intralesional antimoniate pentavalent therapy schedules among New World leishmaniasis studies.

Year, author	Country (origin of patients)	Number of infiltrations	Interval between infiltrations (days)	Treatment duration (interval between the first and the last infiltration in days)
**2016, da Silva**	Brazil	1–6	14	Up to 42
**2016, Soto A**	Bolivian	3	2	5
	Bolivian	5	1 day until the eighth day, with the next application after two days interval	
**2016, Soto B**	Bolivian	5	1 day until the eighth day, with the next application after two days interval	11
**2013, Soto**	Bolivian	3	2	5
**2012, Vasconcellos**	Brazil	1–4	15	Up to 60
**1997, Oliveira-Neto**	Brazil	1–3	14	Up to 21
**1995, Yépez**	Venezuela	3–6 (Mean 4)	7	Up to 50
**1995, Gadelha**	Brazil	4–10 (Mean 5.3)	7 and, with the improvement:10–15	NR

**NR:** no reported

Of the available data, no study described systemic side effect during or after treatment with IL-Sb^v^. Different pain intensities, local hypersensitivity, erythema and edema were the most commonly reported local side effects. Twelve studies (30%) did not report the occurrence of side effects [13, 17, 19, 30, 32, 34, 41–46].

### Methodological quality

For the 27 randomized controlled trials (RCT), concealment of treatment allocation and blinded outcome assessment were performed in 12 (46%) and 7 (30%) studies, respectively. Analysis according to the intention-to-treat principle was performed in six trials (23%) (S2 Table)[18, 37, 39, 40, 47].

A methodological quality assessment of the 14 non-randomized studies using the Newcastle-Ottawa scale indicated that three studies obtained the maximum score corresponding to nine stars, while one studies scored eight stars, one scored seven stars, seven scored six stars and two had a score of five stars (S2 Table). Overall, the studies exhibited moderate methodological quality. Analyzing trim and fill for probability difference, no missing studies were estimated. This is an indicator of a totally symmetric funnel plot and absence of publication bias. The correction of the meta-analysis model parameters, using Trim and Fill method is presented in S1 Fig.

### Global analysis

**Fig 2:**
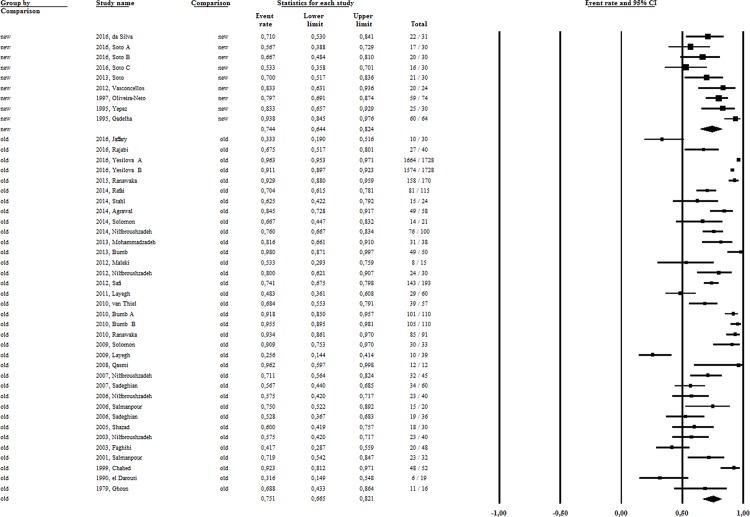
The pooled efficacy rate of Sb^v^ intralesional therapy in New and Old Worlds CL studies.

Our first attempt to address this question was to analyze the effectiveness of intralesional therapy compared to placebo. Only three studies[30, 40, 45], involving 229 patients performed this comparison, two of them in the Americas. The result revealed no difference (OR:1,9), considering the 95% confidence interval (95%CI) ranging from 0,93 to 3,82, based on cure rates of 69.6% (95%CI 17.6–96.1%) and 83,2% (95%CI 66–92.7%) for placebo and IL-Sb^v^, respectively. Applying the strategy of indirect comparison, we then assemble the patients submitted to the same intervention to calculate the pooled efficacy rate of all IL-Sb^v^ study arms. The cure rate retrieved was 75.1% (5679 patients),95%CI 31.6 to 96.3% and high heterogeneity (I^2^ = 93) (Fig 2). The overall IL-Sb^v^ efficacy was 70.3% (95%CI 62.8–76.9%), I^2^ = 93.7 when considering only the 27 randomized controlled trials (1686 patients). Because of the marked differences between *Leishmania* species causing disease in the Old and New World, all analyses were performed separately by geographic region.

### Old World CL studies

**Fig 3:**
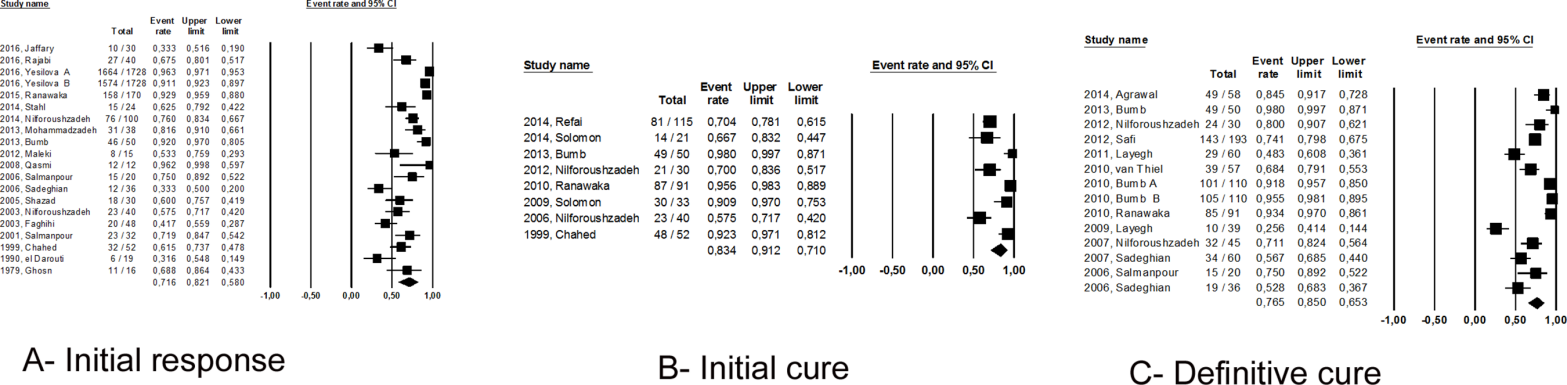
Initial response (A), initial cure (B) and definite cure (C) rates with Sb^v^ intralesional therapy in Old World leishmaniasis studies.

Thirty-three studies (83%) were conducted in the Old World (5336 patients),with an overall intralesional efficacy of 75.1% (95%CI 66.5–82.1%) (Fig 2), without significant difference, by indirect comparison, between the RCTs and non-randomized studies (p = 0.14). Initial response rate and initial and definitive cure rates observed in the Old World studies were 71.6% (95%CI 58–82.1%; I^2^ = 95.8, 4228 patients), 83.4% (95%CI 71–91.2%; I^2^ = 83.4, 432 patients) and 76.5% (95%CI 65.3–85; I^2^ = 89.8, 787 patients), respectively (Fig 3).

Considering that 10 studies (involving 400 patients) have required a negative direct skin smear for cure definition, we evaluated the cure rate grouping the studies according to the cure criterion used: clinical or clinical and parasitological criterion and, as expected, statistical difference can be observed between the rates, which were lower when a negative parasitological examination was required for the definition of cure: 68% (95%CI 51–81%) *versus* 78% (95%CI 68–85%), p = 0.00.

**Fig 4:**
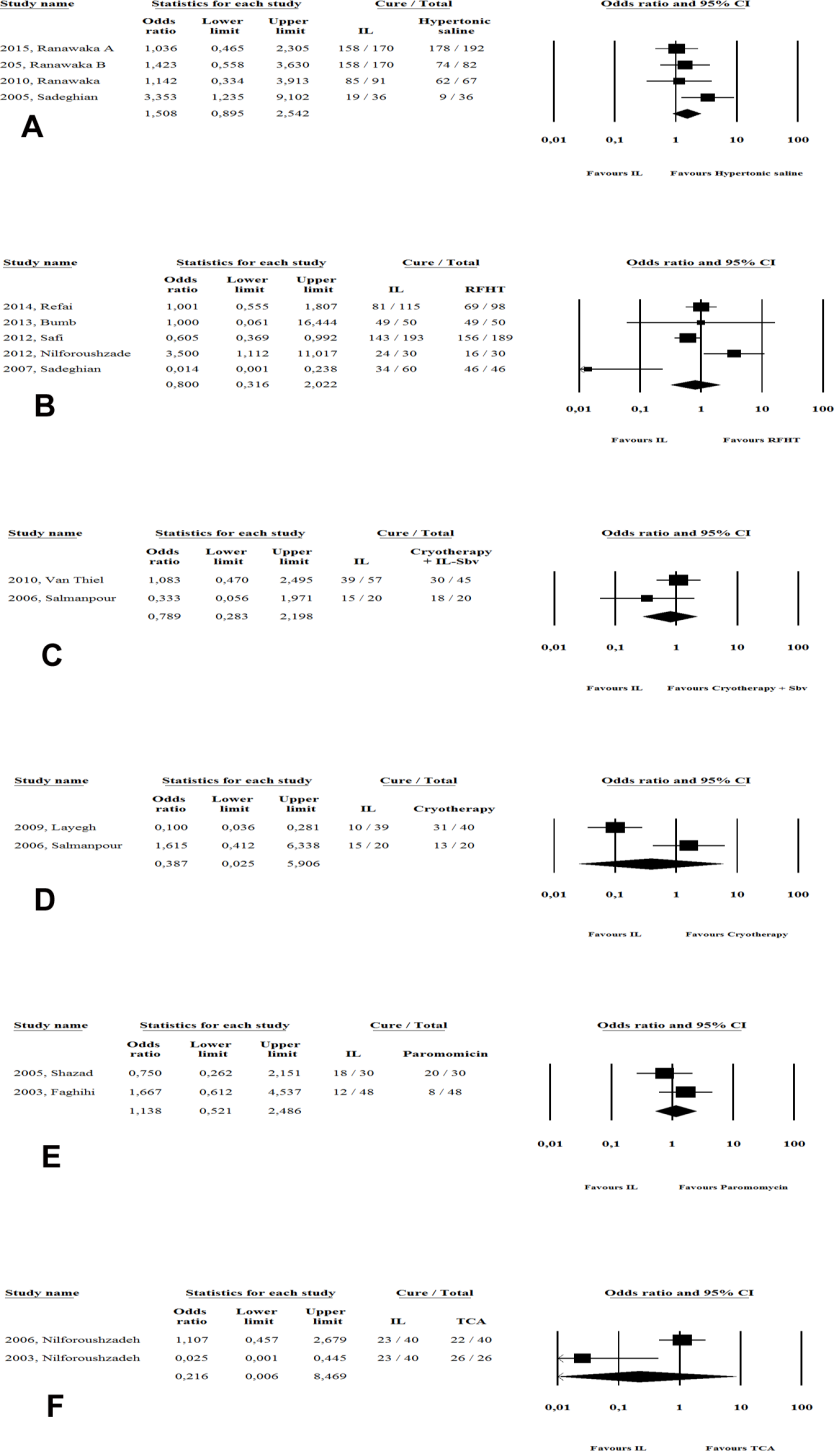
Meta-analysis of studies comparing IL-Sb^v^ directly with topical and physical therapeutic alternatives.

In direct comparisons, no significant difference in efficacy was found between IL-Sb^v^ and 15% paromomycin ointment (p = 0.74; 156 patients) or intralesional hypertonic saline (p = 0.12; 844 patients). Similarly, the other physical methods analyzed produced no different in cure rates compared to IL-Sb^v^, namely for RFHT (p = 0.65;871 patients), TCA (p = 0.41; 153 patients), cryotherapy (p = 0.49;129 patients) or cryotherapy plus IL-Sb^v^ (p = 0.65; 142 patients) (Fig 4).

### New World CL studies

**Fig 5:**
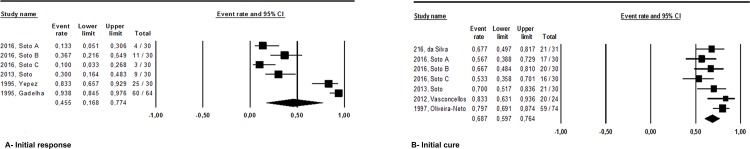
Initial response (A) and initial cure (B) rates with Sb^v^ intralesional therapy in New World leishmaniasis studies.

In total, seven studies were conducted in the Americas. These studies involved 512 participants [15, 16, 30, 39, 40, 48, 49] and had an overall intralesional efficacy of 76.9% (95%CI 66–85%, I^2^ = 69.5), being cure defined clinically in all studies. Three studies (230 patients) were randomized trials [30, 39, 40] conducted by the same investigator but comparing different treatment arms. The efficacy of IL-Sb^v^ was higher in non-randomized studies (282 patients) than in randomized studies: 84.6% (95%CI 72.2–92.1%) *versus* 63.2% (95%CI 52.8–72.6%), p = 0.00. Regarding improvement over time, the early response rate was 68.7%(95%CI 59.7–76.4%, I^2^ = 48, 214 patients), and the initial cure rate was 74.4% (95%CI 64.4–82.4%, I^2^ = 68.9; 249 patients) (Fig 5). A definitive cure rate was reported in only one study [48] as 70.9% (22/31 patients).

MA was used in six studies involving 253 patients [15, 16, 30, 40, 48, 49] and SSG was used in two studies involving 90 patients [39], with a statistically significant difference between them: 82% (95%CI 70–89%) and 61% (95%CI 49–73%), respectively (p = 0.00).

Due to the small number of studies that reported the species of *Leishmania* responsible for infection, it was not possible to analyze the cure according to this informed parameter. However, from the knowledge of *Leishmania* species most prevalent in each geographic region, we performed a deductive analysis of the rate of cure per species. In most studies from Old World, *L*. *tropica* and *L*. *major* are the *Leishmania* species more prevalent and the cure rate for this species was calculated in 73% (95%CI 64–81%; 3250 patients). Three studies were performed in Siri Lanka involving 376 patients, where *L*. *donovani* predominates and the cure rate was estimated in 88% (95%CI 61–96%). In all New World leishmaniasis studies, the predominant specie is *L*. *braziliensis*.

In general, side effects were poorly described. Of the available data, no study described systemic side effects during or after treatment with IL-Sb^v^. Different pain intensities, local hypersensitivity, erythema and edema were the most commonly reported local side effects. Twelve studies (30%) did not report the occurrence of side effects [13, 17, 19, 30, 32, 34, 41–46]. A complete description of the adverse events related to intralesional therapy should include a systematic monitoring of occurrence of pain during infiltration, hypersensitivity reactions, local infections and investigation of all laboratory abnormalities described with parenteral administration.

The studies used different regimens in terms of intervals, length of treatment and number of doses injected (Tables 7 and 8) making it difficult to perform a comparative analysis of efficacy according to the regimen used. Numerous attempts were made to group studies by transforming the therapeutic regimes adopted into dichotomous variables. The characteristic identified as being most related to cure was treatment longer than 14 days (p = 0.00).

**Fig 6:**
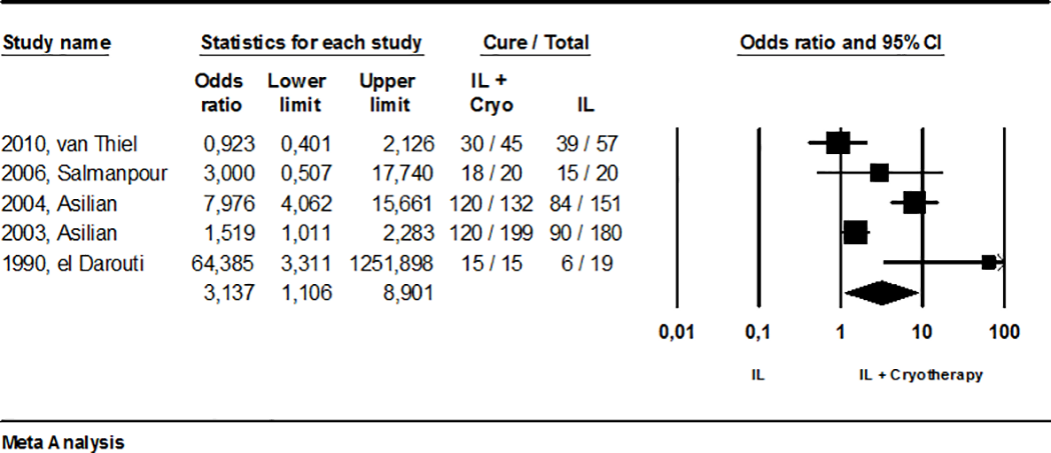
Meta-analysis of studies comparing IL-Sb^v^ directly with IL-Sb^v^ plus cryotherapy: a sensitivity analysis.

Finally, we also looked at studies that used some therapy combined with intralesional infiltration. There are two [31,34] studies involving 260 patients, comparing intralesional approach combined with TCA *versus* antimony intralesional infiltration (OR: 4,2, 95%CI 0.27–63.2, p = 0.3); three studies[14, 22, 27] involving 233 patients and comparing antimony intralesional infiltration combined with cryotherapy *versus* cryotherapy (OR: 3.5, 95%CI 0.80–15, p = 0.09) and finally, three studies involving 176 patients comparing antimony intralesional infiltration combined with cryotherapy *versus* antimony intralesional infiltration alone (OR: 3.7, 95%CI 0.49–27, p = 0.2). In all three of these cases, meta-analysis was unable to detect superiority of the combination over the isolated intervention. It is important to note; however, that the last comparison was made in two other large studies [50, 51] identified in our literature search but not included in our analysis because of the lack of the outcome of interest (number of cured patients). Even that, in order to obtain the most comprehensive analysis as possible and considering that the number of healed lesions have been considered equivalent to the number of patients cured [52–54], we performed a second analysis of the comparison between efficacy of intralesional infiltration combined with cryotherapy in relation to intralesional therapy alone, this time with the two studies previously not considered, in a sensitivity analysis (Fig 6). Using this alternative approach, the cure rate observed with intralesional infiltration and cryotherapy combined (81.8%, 95%IC 62.4–92.4%) was significantly higher when compared with antimony intralesional infiltration alone (53.3%, 95%IC 46.1–66%), OR: 3.14 (95%CI 1.1–8.9), p = 0.03.

## Discussion

The present study is the first systematic review of efficacy of pentavalent antimony intralesional therapy. Gathering a total population of 5679 patients, a global efficacy of 75% (95%CI 68–82%) was observed. This result represents a cure rate similar to that described in the New World for parenteral treatment with antimony [55]and is similar to that found in systematic reviews in the Old World with other local treatments [56–58]. Due to the lack of alternative active chemical principles against *Leishmania*, antimony derivatives remain the first line treatment for all disease forms. Frequent side effects associated with systemic antimonial administration include arthralgia, myalgia, abdominal discomfort, headache, skin eruption and reversible elevation of hepatocellular enzymes [59, 60]. In addition, other severe and life-threatening disorders such as arrhythmias and pancreatitis are reported. Local therapy has emerged as an attractive option for treatment-related toxicity of CL, which is a non-lethal condition. Among the non-systemic therapies, the infiltration of antimony derivatives directly into the lesion has been described for about three decades and was recently included in the recommendations produced by the WHO and PAHO for CL treatment[1].

Sodium stibogluconate and meglumine antimoniate are two widely used antimonial drugs, but the relative efficacies of intralesional MA and SSG therapies in the treatment of CL are not clear. Indirect analysis showed a significantly higher rate of cure in the Old World for treatments performed with IL-SSG (80.3%, 95%CI 70.9–87.2%) compared to IL-MA (71.3%, 95%CI 60.2–80.4%), p = 0.00. This observation contrasts with the better efficacy observed with IL-MA in the sole Turkish study [17], which directly compared the two types of antimony derivatives in intralesional use (82% *versus* 67%). This retrospective study exclusively addressed infections caused by *L*. *major* and *L*. *tropica*. The main explanation for this discrepancy is the difference in the cure criteria adopted. In the original study, the authors assessed cure one month after the first cycle of eight intralesional infiltrations. In our analysis, we considered the clinical outcome after all the infiltrations were performed, in one or two cycles. Unlike in the Old World, indirect analysis including only studies conducted in the Americas has shown superior efficacy with the use of IL-MA compared to IL-SSG (82%; 95%CI 70–89% *versu*s 61%; 95%CI 49–73; p = 0.00). Other studies have showed similar efficacy rates between MA and SSG [55], at least for antimony systemic administration. Thus, these results suggest that different pentavalent compounds could be eligible for intralesional use in different regions of the world. A possible explanation is species-dependent susceptibility, but further evidence is needed to conclude that parasite factors are determinants for the therapeutic response.

Unlike that observed for the spontaneous cure rate, described in up to 60% in the Old World, especially in infections caused by *L*. *major* [61], in contrast to the 6% estimated for *L*. *braziliensis* in the New World in a recent meta-analysis [62], our results reveal no difference in efficacy with intralesional therapy between the Old World and the New World. Even that, different species could have contributed to the high heterogeneity observed in the results. However, we were not able to examine such factors due to an insufficient number of studies presenting cure rates according to the *Leishmania* species.

Despite the justified concern about CL caused by species related to late mucosal involvement, Sb^v^ intralesional long-term efficacy could not be clearly evaluated in this review due to the short period of follow-up adopted in the studies. In most New World leishmaniasis studies, patient follow-up lasted for a maximum of 3 months, and the cure rate at 180 days was presented in only one study [48]. In general, the exact risk of developing ML in an individual with CL cannot be precisely estimated, and statements of late complications associated with non-systemic therapies have been questioned. As evidence of long-term efficacy, mention should be made of the observational study performed by Oliveira-Neto *et al*. [20], where patients treated by Sb^v^ intralesional approach were re-assessed for 10 years, and no cases of relapse or development of mucosal lesions was identified. Another interesting observation was the occurrence of mucosal complications even after systemic antimony treatment [63].

The Sb^v^ intralesional therapy efficacy according to the patients’ age is an aspect worthy of consideration. Children younger than 10 years accounted for 13.5% (6,880) of cases of CL in 2014 in the Americas and may represent more than 30% of cases in some countries [64]. In this review, although 29 studies included children, only four and described the cure rate in this age group exclusively [19, 20, 26, 37]. The pooled efficacy of pediatric studies was no different from the summarized cure rate of studies addressing adults of developing ML (70.5%; 95%CI 32.5–92.2% *versu*s 60.4%; 95%CI 52.7–67.6; p = 0.59). Although current treatment guidelines [1] do not provide distinct recommendations for adults and children, factors related to the disease clinical presentation and host response according to age, as well as variables related to tolerance to local procedures, may influence the therapeutic response and should be considered in future clinical trials.

The description of adverse events was generally insufficient, precluding a detailed analysis of this topic. These events were not systematically reported and were not sufficiently similar to allow meta-analysis. Twelve studies made no mention of the occurrence of adverse events related to IL therapy. Similarly, the short follow-up period, especially in the New World leishmaniasis studies, did not allow the evaluation of the rate of late mucosal complication after Sb^v^ intralesional therapy.

Although we conducted an extensive review, our analysis includes studies with different designs and the main limitation of the present review is the lack of randomized studies comparing Sb^v^ intralesional intervention with parenteral antimony or other therapeutic approaches. It should be noticed that non-randomized studies addressing intralesional approach generally selected patients with a limited number of lesions and no evidence of metastatic, which may represent a selection bias. The direct comparisons available were based on only a few trials using alternative therapies as control arms. This limitation increases the uncertainty of our conclusions, which were necessarily based on limited pooled analyses. However, when there was no or insufficient direct evidence from randomized trials, an adjusted indirect comparison may provide useful or supplementary information on the relative efficacy of competing interventions. This is acritical summary of the evidence currently available, but its validity relies on scarce evidence that may change as larger well-designed studies are conducted. In addition, the overall poor quality in conducting and reporting studies was noted. Specifically, a sensitivity analysis within subgroups was hampered by the wide variety of Sb^v^ intralesional therapeutic schedules used and by the lack of standardization of the measurements used to judge success or failure of a treatment as well as by the diversity of parasite species that cause the disease. This approach was carried out as far as possible to explain the high heterogeneity observed, as seen by the wide range of efficacy observed; rates were as high as 98% [49] and as low as 26% [37]. In addition, to address heterogeneity, we analyzed differences in methodology, patient characteristics and intervention differences that could explain discrepancies by analyzing subgroups of studies grouped by similarity (e.g., design, geographical location, and age of patients) as well as by performing the analysis after the exclusion of outliers. For example, to explore the extent to which study design influenced the results, cure rates obtained by RCTs and non-randomized studies were compared. From a statistical point of view, we performed regression modeling and direct comparisons when possible. All pooled rates were calculated using the Mantel–Haenszel random effects model. Therefore, the pooled cure rates presented here should not be taken as the definitive measure of effectiveness of the intervention, but rather be analyzed in a comparative way putting into perspectives all measures for the several subgroups in order to recognize the various factors influencing the results, in an attempt to get closer to the true.

Few advances can be recognized in leishmaniasis treatment. But two trends can be recognized: the recognition of the inadmissible toxicity associated with systemic antimony derivatives treatment, which has generated a shift of focus towards local therapies and, related to the former, the understanding that probably we need not a single ideal therapy for all CL patients, but rather the most appropriate therapy for each group of patients. In conclusion, the evidence base for Sb^v^ intralesional treatment has many limitations. Addressing these deficiencies in future high-quality clinical trials should be a priority and one of the main conclusions of this review. The confirmation of type of causative *Leishmania* species, a longer follow-up period after local therapies and the assessment of quality of scars are important points to be included during study protocol planning. Another very important point to be evaluated is the efficacy of treatments combining antimony intralesional infiltration with some systemic or even topical therapy. Specifically, the cure rate observed with IL-Sb^v^ and cryotherapy combined are encouraging and may represent a new and promising modality for CL treatment, to be confirmed in new trials.

Current evidence, based mainly on a relatively large body of non-comparative studies support the use of an intralesional Sb^v^ approach as a therapeutic alternative for Old and New World CL. Randomized trials to compare the efficacy of various pentavalent compounds in different world regions are required. This review highlights the need to implement a standardized intralesional technique. New therapeutic studies in CL must be conducted in accordance with current methodological guidance [7] to harmonize and improve the quality of future evidence. An optimal Sb^v^ intralesional schedule should also be established.

## Supporting information

S1 Text Systematic review search strategy. (DOC)

S1 Table Characterisics of excluded studies assessing outcome as number of cured lesions and not as the number of cured patients. (DOCX)

S2 Table Studies’ quality assessment. (DOCX)

S3 Table PRISMAChecklist. (DOC)

S1 Fig Publication bias analysis. (TIF)
