# Supplementary material for: Efficacy of pentavalent antimoniate intralesional infiltration therapy for cutaneous leishmaniasis: A systematic review
Source: PLoS One. 2017 Sep 19;12(9):e0184777. doi: 10.1371/journal.pone.0184777 (PMC5604971; doi:10.1371/journal.pone.0184777)
Supplement: S1 Text — (DOC) [file pone.0184777.s001.doc]

**SEARCH STRATEGY**

The following strategies were used to identify all studies that were conducted in humans and evaluated the efficacy or safety of treatment with intralesional pentavalent antimony compounds in CL from inception through september, 2016.

**A. PubMed:** 265 reports were identified.

((((((("Leishmaniasis, Cutaneous"[Mesh:noexp]) OR ((Cutaneous Leishmaniasis[Title/Abstract] OR "Leishmaniasis, Cutaneous"[Title/Abstract])))) AND (((((("Antimony"[Mesh]) OR "Meglumine"[Mesh:noexp]) OR "Antimony Sodium Gluconate"[Mesh]) OR "Antiprotozoal Agents"[Mesh:noexp])) OR ((Antimony[Title/Abstract] OR Meglumine[Title/Abstract] OR "Antimony Sodium Gluconate"[Title/Abstract] OR "Antiprotozoal Agents"[Title/Abstract] OR "meglumine antimoniate"[Title/Abstract])))))) AND ((((topical OR administration topical)) OR administration, topical[MeSH Terms]) OR (infiltration OR intralesional))).

**B. Lilacs:** 15 reports were identified.

((mh:c03.752.300.500.400 OR "Leishmaniose Cutânea" OR "Leishmaniasis, Cutaneous" OR "Leishmaniasis Cutánea" OR "Cutaneous Leishmaniasis") AND (mh:d01.268.513.124 OR antimônio OR antimony OR antimonio OR mh: d02.033.800.813.550 OR meglumina OR meglumine OR mh: d02.241.081.844.322.060 OR "Gluconato de Antimônio e Sódio" OR "Antimony Sodium Gluconate" OR "Gluconato de Sodio Antimonio" OR mh:d27.505.954.122.250.100 OR antiprotozoários OR "Antiprotozoal Agents" OR antiprotozoarios OR "antimoniato de meglumina" OR "meglumine antimoniate")) AND (infiltração OR infiltración OR infiltration OR intralesional) AND (instance:"regional") AND ( db:("LILACS" OR "IBECS")).

**C. ICTRP:** “leishma*”
