# Supplementary material for: Efficacy of pentavalent antimoniate intralesional infiltration therapy for cutaneous leishmaniasis: A systematic review
Source: PLoS One. 2017 Sep 19;12(9):e0184777. doi: 10.1371/journal.pone.0184777 (PMC5604971; doi:10.1371/journal.pone.0184777)
Supplement: S1 Table — (DOCX) [file pone.0184777.s002.docx]

**Main methodological characteristics of the Old World Leishmaniasis studies**

| Year, Author | Country (cases) | Study arms  (patients) | Prospective/ Comparative | Randomized | Inclusion criteria | Exclusion criteria | Cure criteria for ulcered lesions | Follow-up (months) |
| --- | --- | --- | --- | --- | --- | --- | --- | --- |
| 2013, Khatami | Iran  (83) | MA-IL (26)  MA-IL + non-silver dressing (26)  MA-IL + silver dressing (31) | Yes/Yes | Yes | Parasitologically confirmed diagnosis; otherwise healthy subjects based on medical history, age of 12 to 60 years. | Pregnant or lactating women; duration of lesion > 3 months; number of lesions > 5; ulcer size > 5 cm; history of receiving full course standard treatment; allergy to MA or silver; serious systemic illnesses; participation in any drug trials in the last 60 days; indication for systemic treatment; presence of secondary bacterial infection | Complete healing defined as more than 75% | 7,2 |
| 2010, El-Sayed | Yemen  (30) | SSG-IL (10)  SSG-IL + SSG-IM (10)  SSG-IL + Ketoconazole (10) | Yes/Yes | Yes | NR | Patients who had skin lesions of more than 8 weeks duration, patients who were known to be allergic to antimonial drugs, pregnant or lactating | Complete re-epithelialization, disappearance of edema, induration and other signs of inflammation and a negative direct smear | 6 |
| 2004, Asilian | Iran  (400) | Cryo + MA-IL (100)  Cryo (200)  MA-IL (100) | Yes/Yes | Yes | Parasitologically confirmed diagnosis | Disease duration > 8 weeks, allergy to antimonial, and lactating or pregnant | Complete re-epithelialization, disappearance of edema, induration, and other signs of inflammation, and a negative direct smear. | 6 |
| 2003, Asilian | Iran  (180) | Cryo + MA-IL (40);  Cryo + SSG-IL (40)  MA-IL (100) | Yes/Yes | No | NR | Patients who had first noticed a skin lesion more than 8 weeks previously, who were known to be allergic to antimonial drugs, who were lactating and/or who were pregnant | Complete re-epithelialization, disappearance of edema, induration and other signs of inflammation plus a negative direct skin smear | 6 |
| 2000, Gurei | Turkey  (97) | Cryotherapy (42)  SSG-IL (55) | Yes/Yes | No | Any age or sex, any number of lesions, no pregnancy and no prior treatment | Patients had 7 months or older lesions | Complete healing and disappearance of all clinical features. | 3 |
| 1999, Mujtaba | Pakistan  (96) | MA-IL weekly (49)  MA-IL fortnightly (47) | Yes/Yes | Yes | Parasitologically confirmed diagnosis | >5 lesions and pregnant or lactating. | 100% improvement | 2 |
| 1998, Sharquie | Iraq  (69) | Baghdadin device (54)  SSG-IL (15) | Yes/Yes | No | NR | NR | Marked improvement or total clearances plus a negative direct skin smear | 1.5 |
| 1997, Sharquie | Iraq  (85) | ZnSO4 (19)  NaCl (17)  SSG-IL (18)  No treatment (9) | Yes/Yes | Yes | Parasitologically confirmed diagnosis | Cases of re-infection | Both the marked improvement and total clearance | 1.5 |
| 1996, Tallab | Saudi Arabia  (96) | SSG-IL daily (32)  SSG-IL alternate days (32)  SSG-IL weekly (32) | Yes/Yes | No | Parasitologically confirmed diagnosis | Pregnancy, no prior treatment (intralesionally, orally, or parenterally) | 100% improvement with no relapse after 6 months of follow-up | 6 |
| 1993, Faris | Saudi Arabia  (710) | SSG-IL (710) | Yes/No | No | Typical acute CL with an average duration of symptoms of 3 months | NR | Disappearance of the lesion | 3 |
| 1991, Harms | Syria  (40) | rIFN-y (20)  MA-IL (20) | Yes/Yes | Yes | Up to 3 lesions diagnosed clinically and parasitologically as CL, absence of chronic or acute severe systemic disease | Pregnancy, and no prior antimonial medication | Complete healing, smooth scar and parasites not detectable | 2.5 |
| 1988, Sharquie | Iraq  (60) | SSG-IL (60) | Yes/No | No | Typical acute CL and parasitologically confirmed diagnosis | Cultures which failed to show growth within 3 weeks were considered negative and these lesions | Total clearance of the lesion with parasites not detected in the affected site by smears or culture | 1.4 |

**NR:** no reported **^a^:** number of patients **^b^:** number of lesions **Cryo:** Cryotherapy **Keto:** Ketoconazole **MA-IL:** intralesional meglumine antimoniate **ZnSO4:** zinc sulfate **SSG-IL:** intralesional sodium stibogluconate **NaCl:** Sodium chloride **rIFN-y:** recombinant interferon-gamma **IM**: intramuscular

**Characteristics of the population and outcomes in Old World Leishmaniasis studies**

| Year, Author | Country (cases) | Mean of lesions per patient ± SD | Lesion site ^a,b^  ^a: number of patients^  ^b: number of lesions^ | Study arms (number of patients) •number of lesions | Epithelization rate between 30-73 days, number of lesions (%); | Epithelization rate between 74-100, number of lesions (%) | Epithelization rate between 101-194 days, number of lesions (%) |
| --- | --- | --- | --- | --- | --- | --- | --- |
| 2013, Khatami | Iran  (83) | 1.9 ± 1.2 | NR | MA-IL (26) •45  MA-IL + non-silver dressing (26) •53  MA-IL + silver dressing (31) •60 | 16/45  19/53  20/60 | - | - |
| 2010, El-Sayed | Yemen  (30) | 1.3 | Face (36), upper extremities (4)^b^ | SSG-IL (10) •12  SSG-IL + IM (10) •15  SSG-IL + Keto (10) •13 | 4/12  12/15  11/13 | 7/12  14/15  12/13 | - |
| 2004, Asilian | Iran  (400) | 1,2 | NR | Cryo + MA-IL (100) •132  cryo (200) •210  MA-IL (100) •151 | 120/132 (90.9)  90/210 (57.1)  84/151 (55.6) | - | - |
| 2003, Asilian | Iran  (180) | 1.7 | NR | Cryo + MA-IL (40) •67  Cryo + SSG-IL (40) •65  MA-IL (100) •180 | 60/70 (89.5)  60/65 (92.3)  90/180 (50) | - | - |
| 2000, Gurei | Turkey  (97) | 1,4 | Face (80), upper extremity (42), lower extremity (11)^b^ | Cryotherapy (42) •60  SSG-IL (55) •73 | 46 /60 (77)  • 62/73 (85) | 44 /60 (73)  62/73 (85) | - |
| 1999, Mujtaba | Pakistan  (96) |  | Upper limbs (93), face (64), lower limbs (57), trunk (1)^b^ | MA-IL weekly (49) •111  MA-IL fortnightly (47) •104 | 102/111 (92)  89/104 (86) | - | - |
| 1998, Sharquie | Iraq  (69) | 2.9 | Face (16), trunk (9), upper limbs (72), lower limbs (85) ^b^ | Baghdadin device (54) •146  SSG-IL (15) •36 | 135/146 (92.5)  32/36 (88.9) | - | - |
| 1997, Sharquie | Iraq  (85) | 2.4 | Head and neck (46), upper limb (47), lower limb (55), trunk (3) ^b^ | ZnSO4 (19) •38  NaCl (17) •40  SSG-IL (18) •35  No treatment (9) •38 | 36 /38 (94,8)  34/40 (85)  31/35 (88,6)  38/38 (100) | - | - |
| 1996, Tallab | Saudi Arabia  (96) | 1.3 | Face (85), upper extremity (32), lower extremity (7), neck (4), trunk (1)^a^ |  |  |  |  |
| 1993, Faris | Saudi Arabia  (710) | 1.4 | Cheek (297), lips (189), nose (136), chin (36), forehead (25), eyelid (17), ear (14), upper limbs (238), lower limbs (98)^b^ | SSG-IL (710) •1050 | 756 /1050 (72) | - | - |
| 1991, Harms | Syria  (40) | 1.9 | Upper extremity (40), lower extremity (18), face (10), trunk (1)^b^ | MA-IL (20) •38  rIFN-y (20) • 37 | 29/38 (76)  1/37 (3) | - | - |
| 1988, Sharquie | Iraq  (60) | NR | NR | SSG-IL (60) •130 | 123/130 | - | - |

**NR:** no reported **^a^:** number of patients **^b^:** number of lesions •: number of lesions **Cryo:** Cryotherapy **Keto:** Ketoconazole **MA-IL:** intralesional meglumine antimoniate **ZnSO4:** zinc sulfate **SSG-IL:** intralesional sodium stibogluconate **NaCl:** Sodium chloride **rIFN-y:** recombinant interferon-gamma **IM**: intramuscular
