# Supplementary material for: Efficacy of pentavalent antimoniate intralesional infiltration therapy for cutaneous leishmaniasis: A systematic review
Source: PLoS One. 2017 Sep 19;12(9):e0184777. doi: 10.1371/journal.pone.0184777 (PMC5604971; doi:10.1371/journal.pone.0184777)
Supplement: S2 Table — (DOCX) [file pone.0184777.s003.docx]

**Table 1. The Newcastle-Ottawa Scale (NOS) for assessing the quality of nonrandomized studies**

| **Non RTC studies**  **Year, author** | **Selection** | | | **Comparability** | | | **Assessment of Outcome** | | | **Total Quality score** |
| --- | --- | --- | --- | --- | --- | --- | --- | --- | --- | --- |
|  | Representativeness of treated arm | Selection of the comparative treatment arm (s) | Ascertainment of the treatment regimen | Demonstration that outcome of interest was not present at star of study | Comparability between patients in different treatment arms – main factor: characteristic of lesions | Comparability between patients in different treatment arms – secondary factor: age | Assessment of outcome with independency (record linkage) | Adequacy of Follow up length  (to assess outcome) | Lost to follow up acceptable (less than 10% and reported) |  |
| **2016, Yesilova** | * | * | * | * | * | * | * | * | * | 9 |
| **2014, Agrawal** | * |  | * | * |  |  | * | * | * | 6 |
| **2014, Solomon** | * | * | * | * |  |  | * | * | * | 7 |
| **2013, Mohammadzadeh** | * |  | * | * |  |  | * | * |  | 5 |
| **2010, van Thiel** | * |  | * | * |  |  | * | * | * | 6 |
| **2009, Solomon** | * | * | * | * |  | * | * | * | * | 8 |
| **2008, Qasmi** | * |  | * | * |  |  | * | * | * | 6 |
| **1990, el Darouti** | * | * | * | * | * | * | * | * | * | 9 |
| **1979, Ghosn** | * |  | * | * |  |  | * |  | * | 5 |
| **2016, da Silva** | * |  | * | * |  |  | * | * | * | 6 |
| **2012,**  **Vasconcellos** | * |  | * | * |  |  | * | * | * | 6 |
| **1997, Oliveira-Neto** | * |  | * | * |  |  | * | * | * | 6 |
| **1995, Gadelha** | * |  | * | * |  |  | * | * |  | 6 |
| **1995, Yepez** | * | * | * | * | * | * | * | * | * | 9 |

**Table 2. Quality assessment of randomized controlled trials**

| **Year, Author** | **Double-blind** | **Concealment of treatment allocation** | **Blinding of outcome assessment** | **Intention-to-treat analysis** |
| --- | --- | --- | --- | --- |
| **2016, Jaffary** | No | Yes | No | No |
| **2016, Rajabi** | No | No | No | No |
| **2015, Ranawaka** | Yes | No | Yes | Yes |
| **2015, Refai** | No reported | No reported | No reported | No reported |
| **2014, Stahl** | No | yes | No | Yes |
| **2014, Nilforoushzadeh** | Yes | Yes | Yes | No |
| **2013, Bumb** | No | No | No | No |
| **2012, Maleki** | No | Yes | No | No |
| **2012, Nilforoushzadeh** | Yes | Yes | Yes | No |
| **2012, Safi** | No | yes | No | No |
| **2011, Layegh** | No | No | No | Yes |
| **2010, Bumb** | No | No | No | No |
| **2010, Ranawaka** | Yes | No | Yes | No |
| **2009, Layegh** | No | No | No | Yes |
| **2007, Nilforoushzadeh** | Yes | Yes | Yes | No |
| **2007, Sadeghian** | Yes | Yes | Yes | No |
| **2006, Nilforoushzadeh** | No | No | No | No |
| **2006, Salmanpour** | No | No | No | No |
| **2006, Sadeghian** | No | No | No | No |
| **2005, Shazad** | No | No | No | No |
| **2003, Nilforoushzadeh** | no | No | No | No |
| **2003, Faghihi** | No | Yes | No | No |
| **2001, Salmanpour** | No | Yes | No | No |
| **1999, Chahed** | Yes | No | Yes | No |
| **2016, Soto** | No | Yes | No | Yes |
| **2013, Soto** | No | Yes | No | Yes |
