# Supplementary figures and images for: Efficacy of pentavalent antimoniate intralesional infiltration therapy for cutaneous leishmaniasis: A systematic review
Source: PLoS One. 2017 Sep 19;12(9):e0184777. doi: 10.1371/journal.pone.0184777 (PMC5604971; doi:10.1371/journal.pone.0184777)

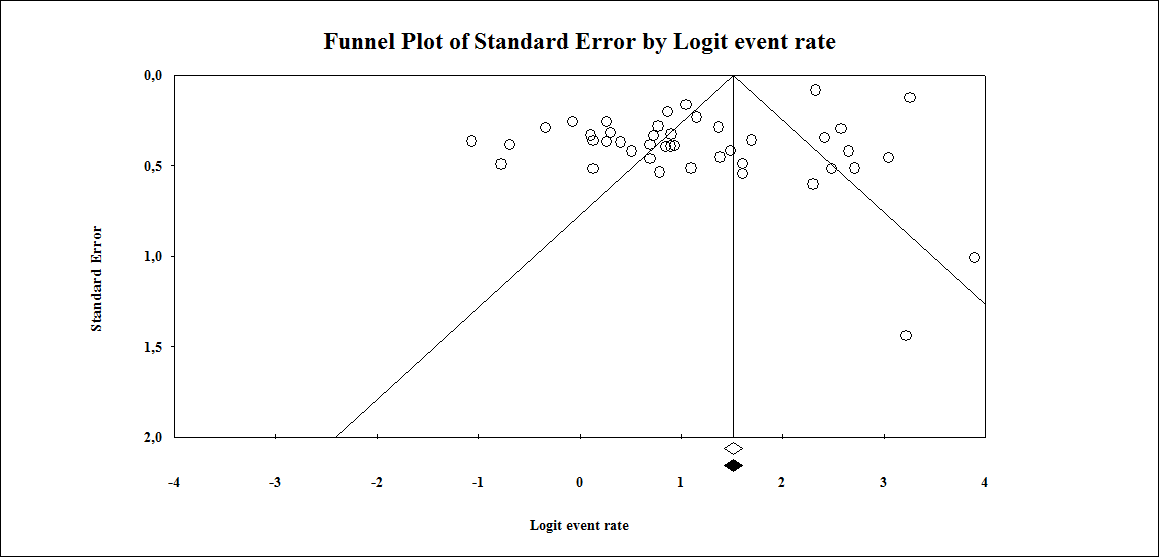

Supplement: S1 Fig — (TIF) [file pone.0184777.s005.tif]
